# Simulation-based optimization analysis of passenger flow organization in metro interchange stations using AnyLogic

**DOI:** 10.1038/s41598-026-41719-5

**Published:** 2026-03-07

**Authors:** Yuhang Tian, Guowei Jin, Shizheng Lu, Wenlong Ma, Nan Li, Guangtao Cao, Wenjie Wang

**Affiliations:** 1 School of Traffic and Transportation, Shijiazhuang Tiedao University, Shijiazhuang, 050043 China; 2 Hebei Key Laboratory of Traffic Safety and Control, Shijiazhuang, 050043 China; 3 Multimodal Transport Department, China Railway Express Co., Ltd.,, Beijing, 100055 China

**Keywords:** Metro, Passenger flow organization, Peak periods, Social force model, Guiding signs, Anylogic, Engineering, Mathematics and computing

## Abstract

In large-scale metro interchange stations, significant passenger flow volumes during peak hours are prone to induce pedestrian congestion phenomena, presenting operational challenges. Taking Metro S transfer station as the research object, this study constructs an AnyLogic pedestrian simulation platform based on the social force model to simulate the current passenger flow conditions in the station concourse area, identifying key bottleneck zones. By employing an optimization method that integrates passenger flow guidance with coordinated allocation of equipment resources, a dual-path passenger flow diversion mechanism is designed to alleviate congestion caused by intersecting passenger flow lines. The optimization results demonstrate that this approach can effectively mitigate peak-hour congestion while reducing passenger walking time and improving throughput efficiency. This offers decision support for passenger flow management in large metro transfer stations.

## Introduction

Currently, urban rail transit systems in major cities have taken shape, and an increasing number of people are choosing subways as their preferred mode of transportation. Due to the subway’s advantages, such as speed, punctuality, and convenience, the proportion of railway passengers opting for subway connections to reach their destinations is steadily rising. For large railway hub stations, the passenger flow capacity of subways significantly surpasses that of other transfer modes. However, the increase in subway passenger volume also heightens the complexity of managing passenger flow within subway stations. Enhancing passenger flow efficiency and preventing bottlenecks are critical issues that require focused attention.

Improvements can be made by optimizing the layout of various facilities within subway stations. Ding Bo et al.^1^ conducted simulation analyses of subway station equipment layouts and proposed optimization schemes based on passenger flow distribution density and related findings. Sun Chen^2^ addressed passenger flow bottleneck issues in existing subway stations by proposing adjustments to the quantity and arrangement of facilities. Zhang et al.^3^ performed simulation modeling of passenger entry, exit, and transfer processes, suggesting measures to rationally arrange transfer points to alleviate passenger congestion. Xie Hui and Zhao Xia^4^ optimized passenger flow congestion in subway stations by adjusting the positioning of escalators. Additionally, improvements can be made through passenger flow streamline optimization. Suo et al.^5^ proposed an optimization method based on pedestrian walking behavior to enhance passenger transit efficiency in subway stations and validated the effectiveness of the proposed scheme. Other studies have explored multifaceted improvements. Zhan Yinxia et al.^6^ conducted simulation analyses of subway stations to identify bottleneck areas of passenger flow congestion, optimizing both pedestrian streamlines and station facility layouts, which reduced pedestrian density in congested zones. Liu Hanying^7^ investigated passenger flows in subway station concourses and platforms, addressing bottleneck issues through optimized equipment allocation and management measures, significantly improving station fluency and comfort. Ni et al.^8^ proposed rational optimization measures for passenger flow congestion in simulation models, focusing on facility utilization rates and passenger flow organization based on flow data. Furthermore, studies have explored the impact of guidance methods on enhancing passenger flow organization efficiency. Xiong et al.^9^ mitigated passenger flow bottlenecks by implementing guidance signage, improving the transit efficiency for entry, exit, and transfer flows. Tang Ziyuan et al.^10^ examined the influence of subway signage positioning on passenger evacuation, enhancing evacuation efficiency by adjusting dynamic signage locations. Huo Liqun and Xu Liangjie^11^ designed various color combinations for guidance signage, evaluating their effectiveness to identify combinations that effectively improve station transit efficiency. Wei Yuhao et al.^12^ identified bottleneck areas in subway station platform and concourse layers, proposing adjustments to guidance methods and train arrival schedules to reduce passenger dwell times in these zones.

Although numerous studies have investigated passenger flow organization in metro stations, most have adopted a single-dimensional optimization perspective. Approaches focusing on facility layout adjustment have indeed alleviated localized congestion to some extent; however, they often neglect the behavioral heterogeneity of passengers and their adaptive responses to spatial guidance information. Conversely, studies emphasizing signage optimization typically assume fixed facility layouts and fail to capture the interactive relationship between information guidance and physical infrastructure. Consequently, there remains a lack of an integrated analytical framework capable of characterizing the coupling mechanisms between facility configuration and behavioral guidance under high-density passenger flow conditions. Therefore, it is essential to develop a collaborative optimization mechanism that systematically coordinates signage strategies with facility allocation. To address this gap, this study proposes a “guidance-facility” synergistic optimization framework, which integrates behavioral guidance and spatial resource configuration within a simulation-based analytical model, thereby enhancing the robustness and practical applicability of passenger flow management in metro interchange stations.

## Materials and methods

### AnyLogic subway station passenger flow simulation method

The implementation of passenger flow simulation in subway stations primarily utilizes the Pedestrian Library in AnyLogic, which is specifically designed for simulating real-world pedestrian flows, enabling the creation of models for pedestrian buildings or streets^13^. The simulation principle of the Pedestrian Library is mainly based on the concept of the Social Force Model, first proposed by Helbing and Molnár in 1995^14^. The core principle of this model involves simulating pedestrian movement decisions in various scenarios by introducing “social forces.” Pedestrian walking behavior is primarily influenced by three forces in the Social Force Model: driving force, repulsive force, and attractive force.

The driving force reflects a pedestrian’s intention to move toward a destination, with its direction pointing toward the target and its magnitude proportional to the difference between the desired speed and the current speed. This force prompts pedestrians to accelerate or decelerate to approach their target. The formula is expressed as follows:

1 $$f_{i}^{d}=\frac{{v_{i}^{0} - {v_i}}}{\tau }$$

In the formula: $v_{i}^{0}$ represents the desired speed of pedestrian *i*,${v_i}$ denotes the current speed, and $\tau$is the time constant for speed adjustment by pedestrian.

The repulsive force prevents collisions between pedestrians or with obstacles, increasing as the distance decreases, ensuring pedestrians maintain a safe distance to avoid physical contact or psychological discomfort. The formula is expressed as:

2 $$f_{{ij}}^{r}={A_i}\exp (\frac{{{r_{ij}} - {d_{ij}}}}{{{B_i}}}){n_{ij}}$$

In the formula: ${A_i}$ and ${B_i}$ are model parameters controlling the strength and range of the repulsive force. ${r_{ij}}$ represents the sum of the radii of pedestrian *i* and another pedestrian or obstacle *j*, ${d_{ij}}$denotes the distance between pedestrian *i* and another pedestrian or obstacle *j*, and ${n_{ij}}$ is the unit vector pointing from pedestrian *i* to another pedestrian or obstacle *j*.

The attractive force describes a pedestrian’s tendency to be drawn toward certain targets (e.g., exits or points of interest), with its direction pointing toward the attraction source and its magnitude determined by the strength of the target’s attractiveness, influencing the pedestrian’s path choice. The formula is given by:

3 $$f_{i}^{a}={C_i}\frac{{r_{i}^{a} - {r_i}}}{{||r_{i}^{a} - {r_i}||}}$$

In the formula: ${C_i}$ represents the attractiveness strength parameter, $r_{i}^{a}$ denotes the position of the attraction source, and ${r_i}$ indicates the current position of pedestrian *i*.

This study’s optimization problem is formulated within a simulation-based scenario optimization framework, rather than a conventional analytical optimization model. Within this framework, the decision variables include the number and spatial layout of active gate groups, as well as the configuration strategies of directional signage; the optimization objectives are to minimize passenger walking time and to reduce passenger density in critical areas; and the constraints are primarily determined by the existing station spatial layout and the fixed passenger demand during peak hours.

It should be noted that the optimal strategy is not obtained by solving a closed-form mathematical optimization problem, but rather by constructing and comparatively evaluating multiple simulation scenarios, through which the passenger flow organization strategy with the best overall performance is identified.

### Subway station simulation modeling

#### Station overview and passenger flow characteristics analysis

**Fig. 1:**
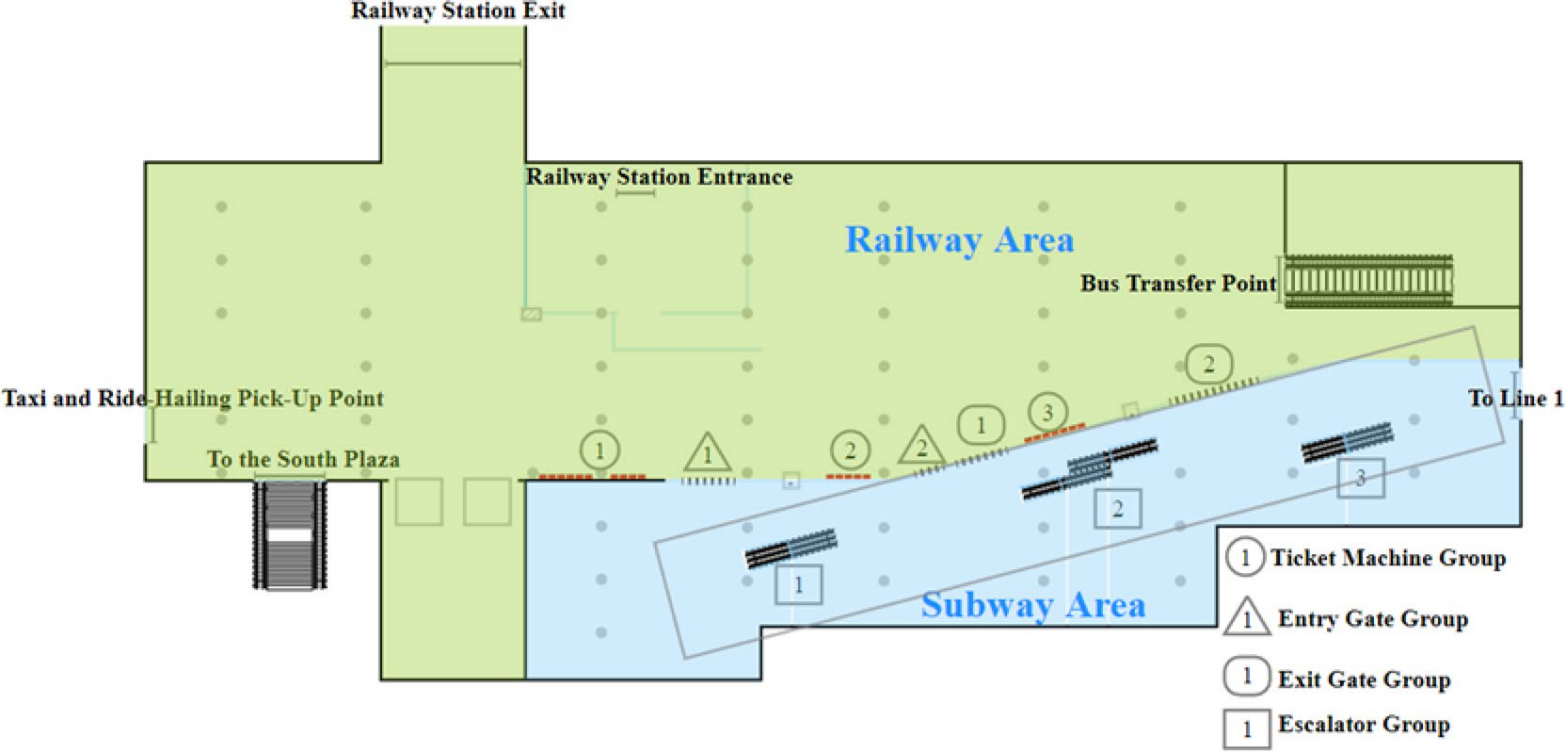
Floor layout of facilities in the subway station concourse.

Station S is a transfer station for Lines 1 and 3 of the H City subway system and serves as a critical subway hub connecting to the city’s railway station. As the passenger flow at Station S primarily originates from arrivals at the railway station, congestion at the entry gates is prone to occur during peak periods of arriving passenger flow, resulting in significant passenger flow pressure. Based on on-site investigations, a simplified layout plan of the concourse level facilities at Station S of the H City subway was developed, as shown in Fig. 1. This study focuses on analyzing the behavior of passengers entering the subway during peak periods of railway passenger arrivals. For analytical simplicity, potential delays induced by cross-flows between metro passengers and those accessing the South Plaza, bus transfer points, and ride-hailing or taxi pick-up areas are not explicitly modeled.

This study assigned numbers to each group of equipment. Ticket Machine Group 1 consists of 10 self-service ticket machines, Ticket Machine Group 2 consists of 5 self-service ticket machines, and Ticket Machine Group 3 consists of 7 self-service ticket machines, though Group 3 is not in use. Additionally, Entry Gate Group 1 and Group 2 have 7 and 4 gates, respectively, while Exit Gate Group 1 and Group 2 have 7 and 12 gates, respectively. Escalator Groups 1, 2, and 3 all serve as passageways to the Line 3 platform layer, with Escalator Group 1 operating exclusively in the downward direction, while Escalator Groups 2 and 3 each have one escalator operating in the upward direction and one in the downward direction.

Based on on-site surveys, the peak hourly inbound passenger flow at Station S is approximately 4,000 passengers per hour, with the flow distribution to Lines 1 and 3 roughly in a 4:6 ratio. The outbound passenger flow is approximately 2,000 passengers per hour, with the flow distribution from the two subway lines mirroring the inbound flow ratio. The high passenger volume easily creates bottlenecks in subway transfer capacity, necessitating improvements to address this congestion.

#### Pedestrian simulation operation process

**Fig. 2:**
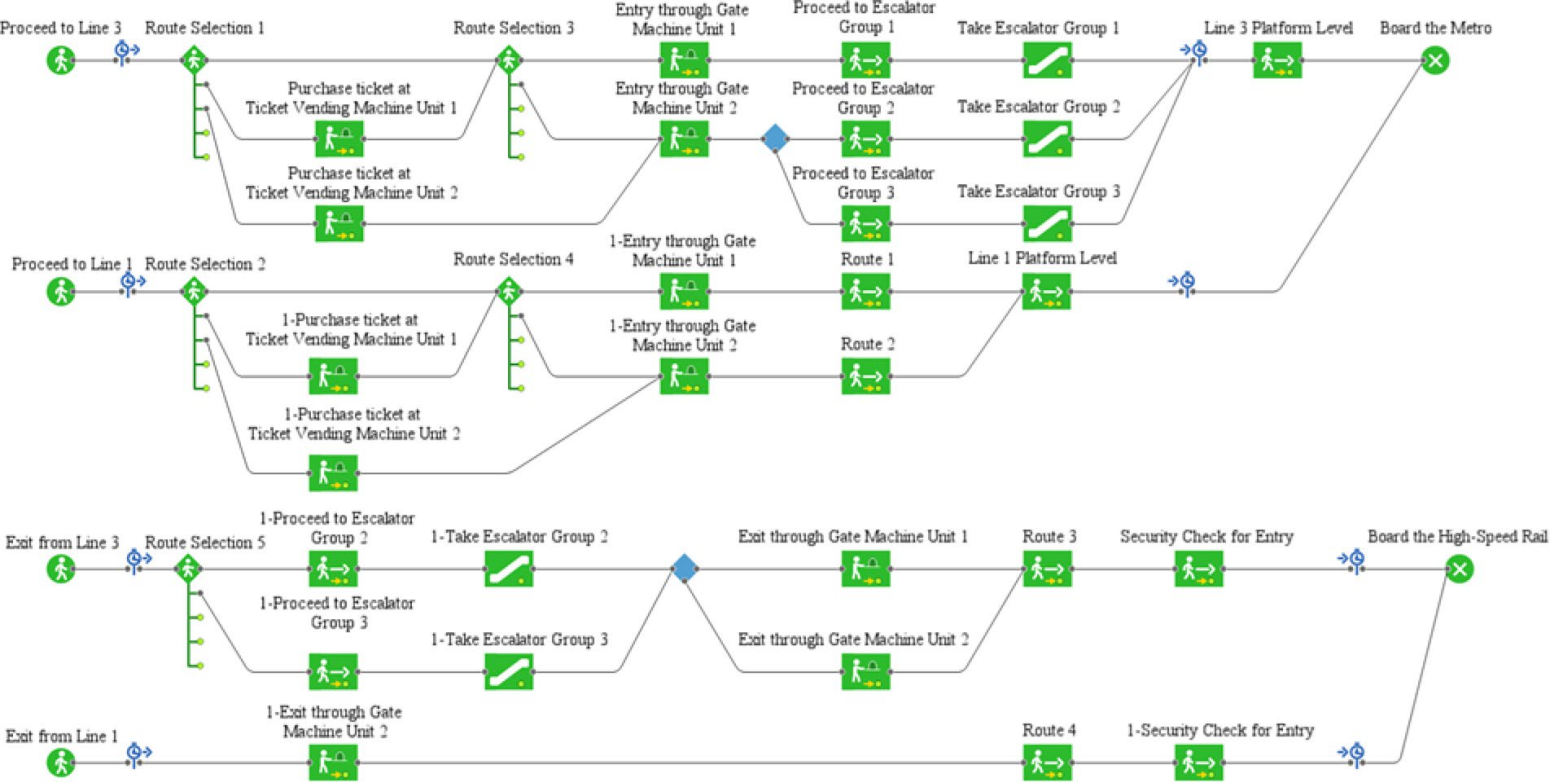
Passenger boarding logic framework diagram.

At H City Railway Station, passengers exiting the railway station are not required to undergo security checks when proceeding to Metro Station S for transfer. The passenger entry process is as follows: passengers exit the railway platform through the railway exit gate into the railway area of the concourse, then proceed to the metro for transfer. Depending on their needs, passengers may choose to purchase tickets at self-service ticket machines before heading to the metro entry gates to access the metro concourse level. Finally, they use escalators or other facilities to reach the platform level of their intended metro line and queue for boarding. Based on the passenger entry process, analyze the logical relationships between the entry and exit procedures for Lines 1 and 3 and the associated facilities. Using the Pedestrian Library and Process Modeling Library in AnyLogic software, construct a logical framework diagram for the passenger entry process, as shown in Fig. 2.

#### Parameter settings

The model includes parameters for pedestrians and service facilities. The pedestrian arrival rate represents the number of pedestrians generated by the source. The initial speed is the walking speed upon entry, and the comfortable speed is the steady-state walking speed. Pedestrian speed is affected by factors such as luggage, cognitive level, passage conditions, and familiarity with the environment. As most inbound passengers come from the high-speed railway station, many carry luggage, which reduces their speed. Pedestrians are classified into two groups—“with luggage” and “without luggage”—in a ratio of approximately 7:3, with the walking speed of the former set to 70% of the latter. Facility parameters include the delay times for ticket purchasing and gate passage, and the operating speed of escalators. Based on the Technical Code for Urban Rail Transit^15^ and field observations, parameters were configured as shown in Tables 1 and 2. Sensitivity analysis showed that moderate parameter variations had little effect on model outputs, confirming the reasonableness and stability of the parameter settings.

**Table 1:** Pedestrian parameter settings.

Module	Parameter name	Parameter value	Unit
Pedestrian Source for Line 1	Arrival Rate	PedSource(1600)	persons/h
Pedestrian Source for Line 3	Arrival Rate	PedSource(2400)	persons/h
Subway Entry Pedestrian Speed	Comfortable Speed	uniform(0.6, 1.0)	m/s
	Initial Speed	uniform(0.3, 0.7)	m/s
Outbound Subway Pedestrian Speed	Comfortable Speed	uniform(0.8, 1.2)	m/s
	Initial Speed	uniform(0.3, 0.7)	m/s

**Table 2:** Service facility parameter settings.

Service facility	Parameter name	Parameter value	Unit
Self-Service Ticket Machine	Delay Time	uniform(20.0, 50.0)	s
Entry Gate	Delay Time	uniform(3.0, 5.0)	s
Escalator	Operating Speed	0.65	m/s

#### Evaluation metrics

To better assess the effectiveness of optimization schemes in improving the current conditions of the subway station, the average queue length at service facilities, passenger flow density at facility locations, and average pedestrian travel time are selected as evaluation metrics. These metrics evaluate the optimization schemes from two perspectives: the smoothness of passenger movement and the congestion levels at station facilities.

1. Average queue length

The average queue length refers to the average number of passengers waiting to be served at each ticket machine group and entry gate group, primarily used to indicate whether the number of gates is sufficient. A higher average queue length suggests lower service efficiency for the corresponding facility. The formula for calculating the average queue length for a service facility group is as follows:

4 $${P_i}=\frac{{\sum\limits_{{j=1}}^{n} {{p_j}} }}{n}$$

In the formula: ${p_j}$ represents the queue length at the *j* gate or ticket machine in the *i* facility group; *n* denotes the total number of gates or ticket machines in the *i* facility group.

(2) In-station passenger flow density

In-station passenger flow density refers to the density of passengers at various service facilities, primarily used to indicate the service level of the corresponding facility and whether congestion occurs as passengers pass through it. A higher passenger flow density suggests greater service pressure on the facility, which is likely to cause passenger flow bottlenecks. The formula for calculating the passenger flow density at a service facility group is as follows:

5 $${\rho _i}=\frac{{{M_i}}}{{{S_i}}}$$

In the formula: ${M_i}$ represents the number of passengers in the selected region around the *i* facility group; S_*i*_denotes the area of the selected region around the *i* facility group.

(3) Average travel time

The average travel time represents the total time a passenger spends in the model, including walking time, service time, and queuing time. It reflects the overall flow efficiency of the system—a longer average travel time indicates lower circulation efficiency. The formula is as follows:

6 $$T=\frac{L}{V}+{T_s}+{T_q}$$

In the formula: *L* represents the distance a passenger travels within the model; *V* denotes the passenger’s walking speed within the model; ${T_s}$ indicates the time required for a passenger to receive services; ${T_q}$ represents the delay time due to queuing for services.

## Results

### Current state simulation results and analysis

**Fig. 3:**
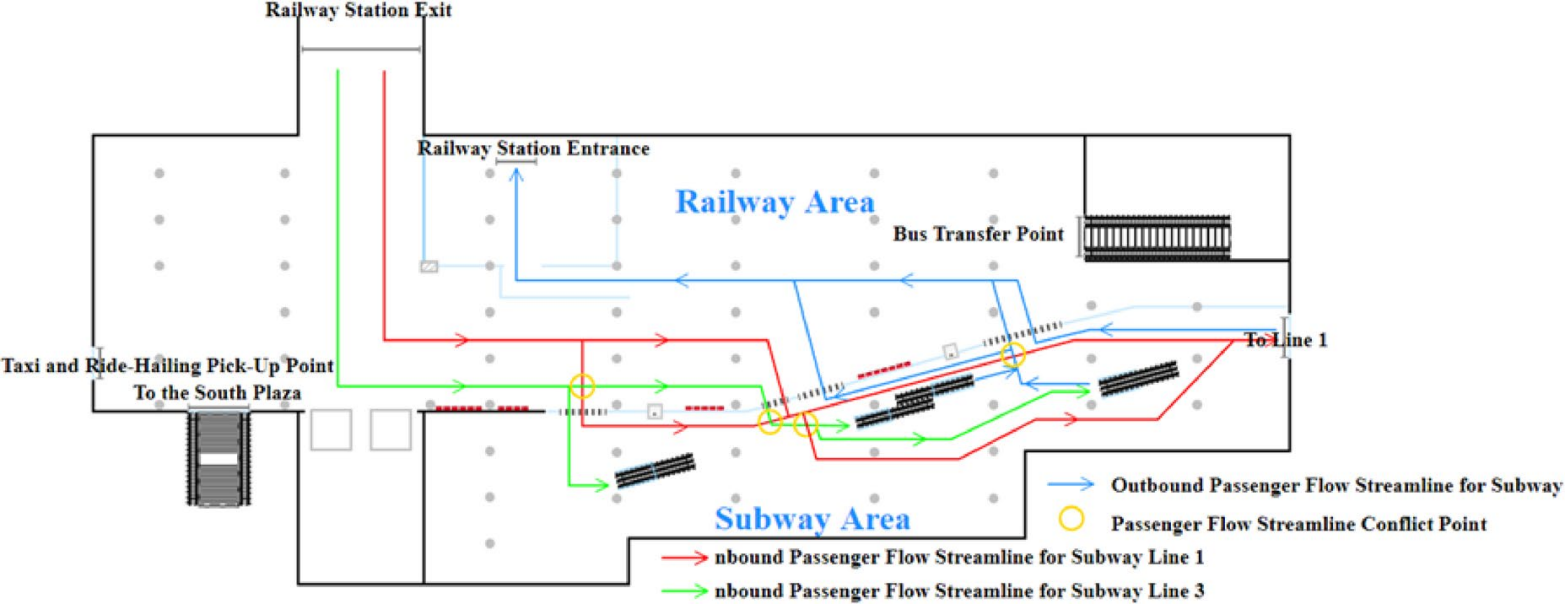
Pre-optimization passenger flow distribution schematic.

The passenger flow streamline distribution at Subway Station S before optimization is shown in Fig. 3. As illustrated, conflicts arise at multiple points among passengers heading to Lines 1 and 3, as well as those entering and exiting the subway station. These conflicts are particularly pronounced in the subway area, leading to intersecting and chaotic passenger flow streamlines. Additionally, the limited space in the subway area exacerbates the impact of these conflicts on passenger transit efficiency.

**Fig. 4:**
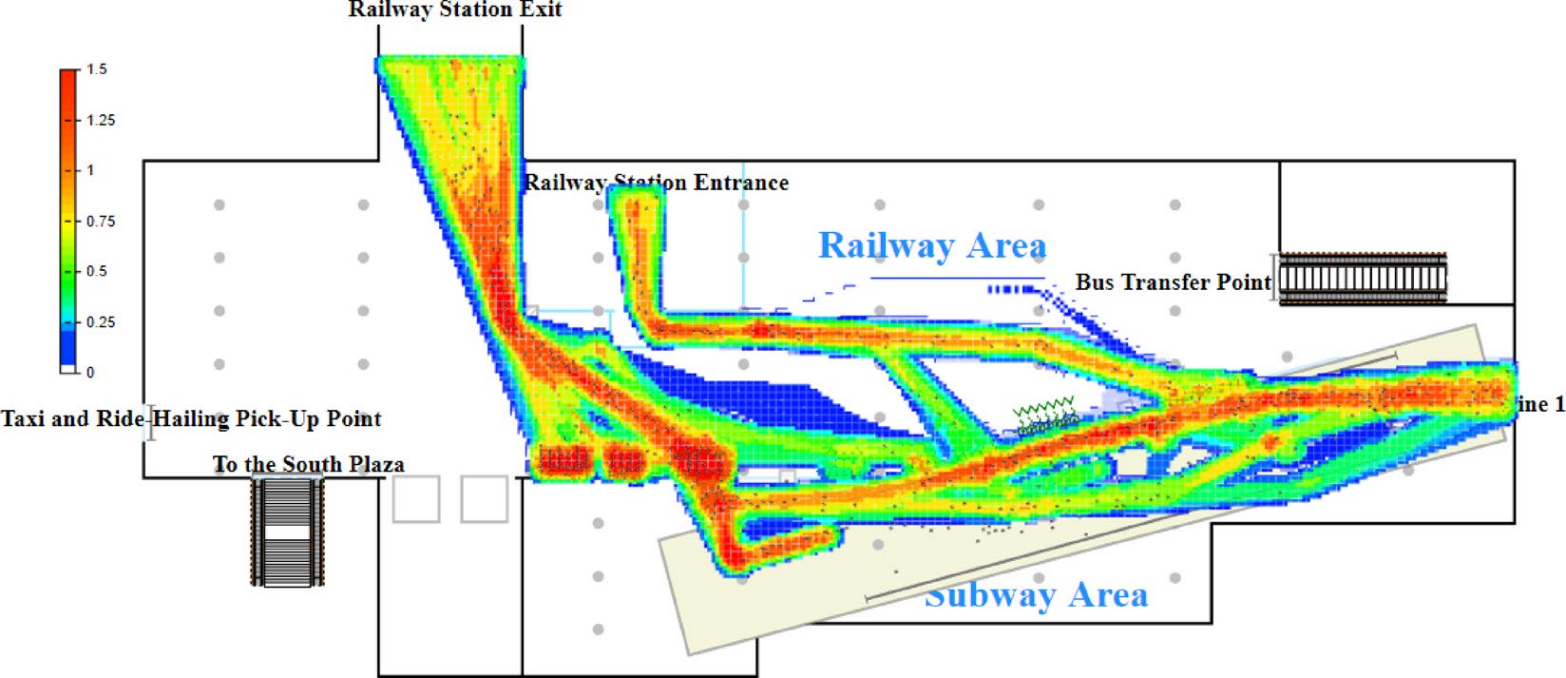
Pedestrian density map before optimization.

Based on the established logical model and the configured parameters, a simulation is conducted in AnyLogic with a one-hour time cycle (Fig. 4). Pedestrian density maps and the queue lengths at service facilities are used as indicators to identify passenger flow bottlenecks within the station. The pedestrian density map obtained from the simulation is shown in Fig. 4.

The simulation results indicate significant passenger queuing and congestion at Ticket Machine Group 1 and Entry Gate Group 1 with passenger flow densities of 1.41 and 1.09, respectively. According to the service level standards proposed by Fruin and the International Air Transport Association (IATA)^6^, these facilities operate at service levels F and E, respectively, indicating severe congestion. This congestion increases passenger travel time and significantly impairs transit efficiency (Fig. 4).

The primary reason is that Entry Gate Group 1 is located closest to the railway station’s exit, and the guidance signage directs passengers bound for Metro Lines 1 and 3 to enter through this area. Consequently, most passengers unfamiliar with the concourse layout—approximately 88.2% of all inbound passengers—choose to enter the metro via Entry Gate Group 1. In addition, the entry gates at Station S are not integrated with commonly used mobile payment applications, requiring passengers to download a dedicated app for QR-code access. As a result, some time-constrained or less tech-savvy passengers opt to purchase tickets at self-service machines instead. Field observations and official statistics indicate that about 28% of inbound passengers buy tickets from self-service machines, with the majority choosing the nearby Ticket Machine Group 1. In contrast, the utilization of Entry Gate Group 2 and Ticket Machine Group 2 remains low, leaving large portions of the concourse area underused and leading to inefficient use of station facilities and space.

### Establishment and analysis of optimization schemes

Based on the analysis of the current passenger flow conditions in the subway station, improvement measures are proposed from two perspectives: in-station facilities and guidance signage.

#### Optimization of in-station facilities

To address the issues of passenger flow streamline conflicts and uneven space utilization within the subway station, the following measures are proposed: Eight gates from Exit Gate Group 2 were converted into Entry Gate Group 3. The remaining gates were retained as Exit Gate Group 2, and three gates were transferred from Exit Gate Group 1 to Exit Gate Group 2. In addition, Ticket Machine Group 3 was activated for operation.

#### Optimization of guidance signage

**Fig. 5:**
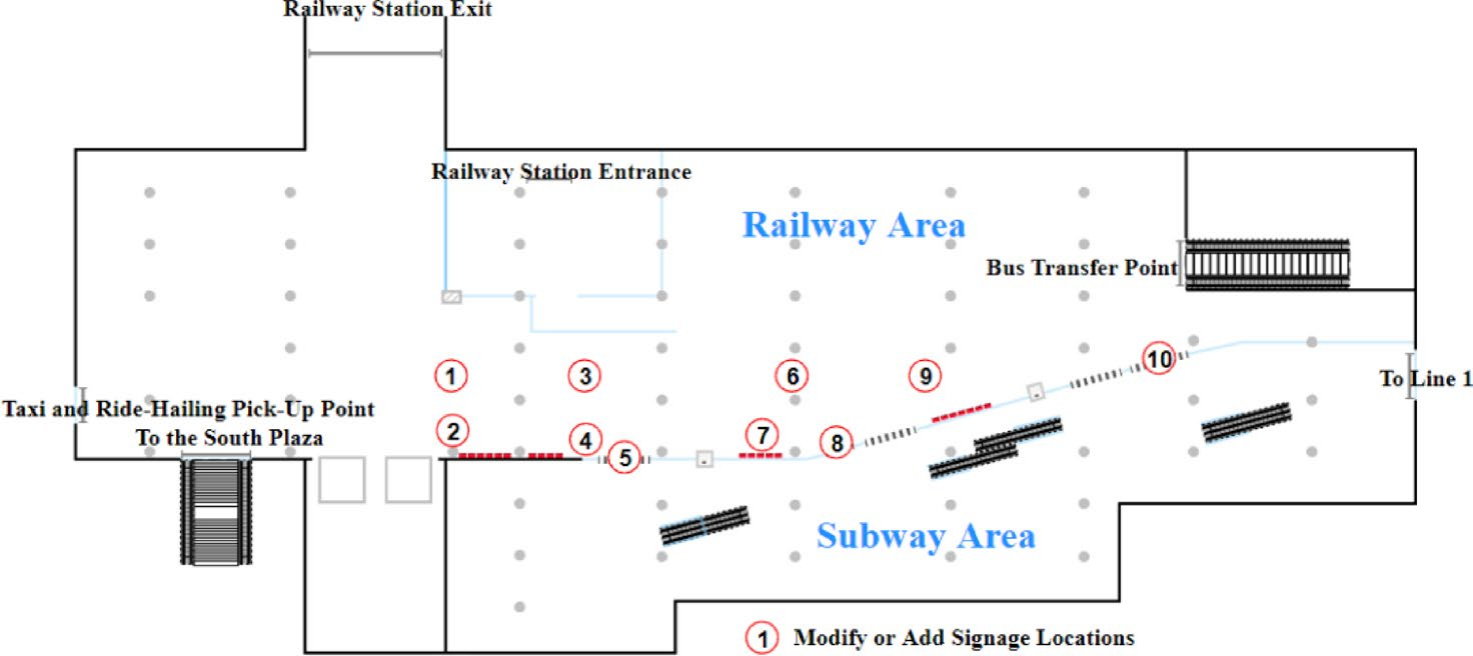
Changes or additions to signage locations in the subway station.

Adjustments are made to the content and placement of signage within the subway station where signage is inappropriate or absent, with corresponding locations marked as shown in Fig. 5.

The selected locations in the figure are primarily at corners of passenger walking paths or at points prone to causing directional confusion. Among the 10 signage locations, positions 1, 3, 5, 6, 8, 9, and 10 utilize overhead signage, while positions 2, 4, and 7 employ freestanding signage or manual guidance. Additionally, the instantaneous memory capacity distribution for passengers across different signage groups follows a normal distribution, with the highest memory accuracy observed for groups containing four information units. As the number of information units increases, memory accuracy declines significantly^16^. Therefore, signage content design should prioritize four information units, with a maximum of six information units per signage. At key decision nodes within the station, passengers are assumed to choose among alternative routes. Following previous studies, the probability of passengers complying with directional signage guidance is set to 70%, while the remaining passengers follow their habitual or shortest paths. This assumption is implemented by assigning route choice probabilities to passengers at each decision node in the simulation.

In Fig. 5, the signage functions are as follows: at position 1, signage directs passengers to the subway station for ticket purchase and boarding; at positions 3 and 6, signage guides passengers heading to Line 1 to proceed straight and those heading to Line 3 to enter on the right; at positions 5 and 8, signage directs passengers to board Line 3; at position 10, signage guides passengers to board Line 1; at position 9, signage instructs passengers heading to Line 1 to purchase tickets on the right and proceed straight to the entry gates for boarding. Additionally, at positions 2 and 7, signage with the message “Passengers heading to Line 1, please purchase tickets at the ticket machines ahead” is added to prevent passengers for both lines from purchasing tickets at the same ticket machines. At position 4, signage with the message “Passengers heading to Line 3 can also proceed 20 meters ahead to enter” is added to guide Line 3 passengers to use Entry Gate Group 2, thereby alleviating the transit pressure on Entry Gate Group 1.

### Comparison of evaluation metrics before and after optimization

**Fig. 6:**
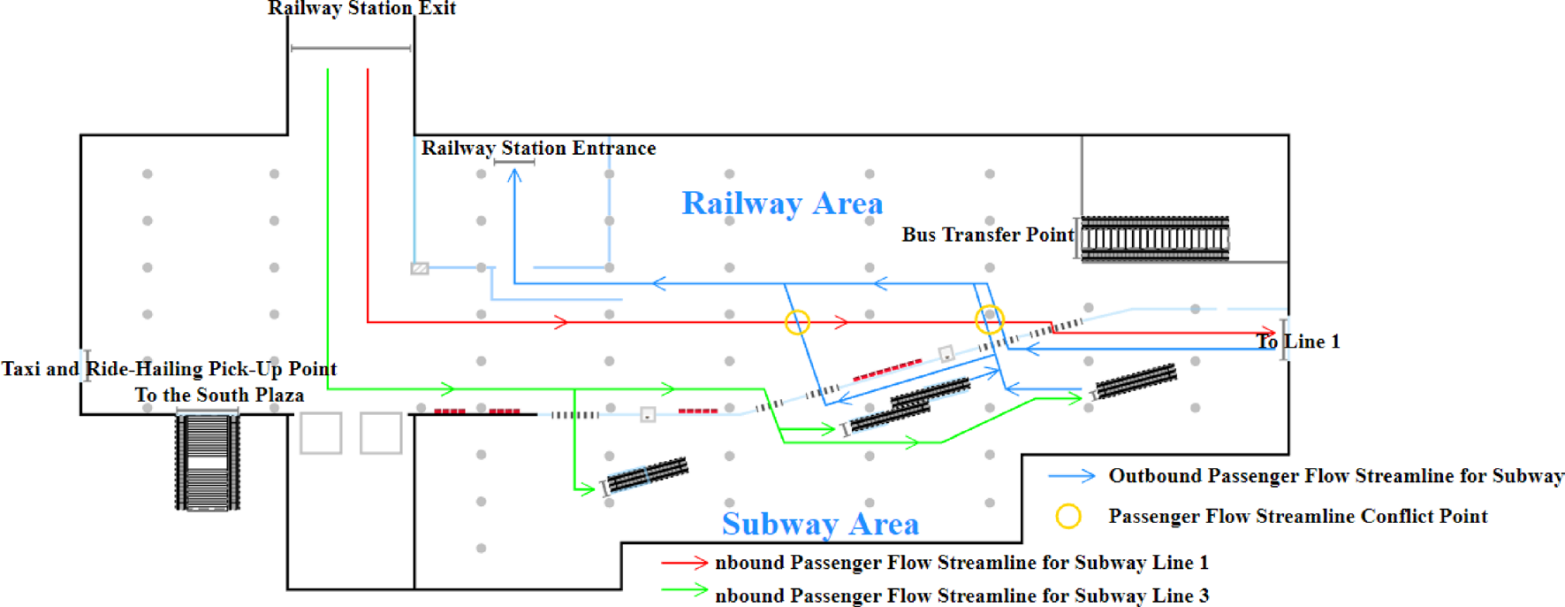
Optimized passenger inbound flow diagram.

**Fig. 7:**
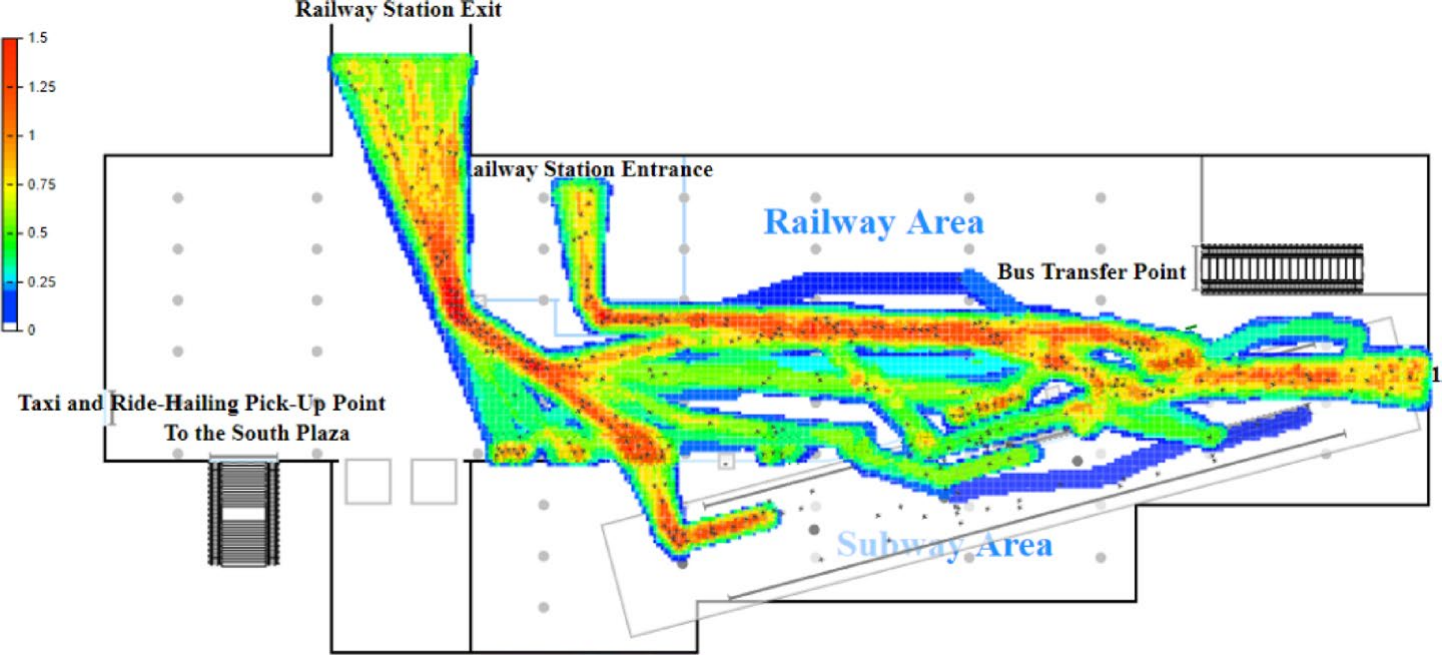
Optimized pedestrian density map.

After optimizing the in-station facilities and guidance signage, passenger flows heading to Line 1 and Line 3 are separated, avoiding streamline conflicts in the confined subway area. This results in clearer passenger flow streamlines, significantly enhancing streamline smoothness and passenger transit efficiency. The optimized passenger flow streamline diagram is shown in Fig. 6. A simulation of the optimized model yields the pedestrian density map presented in Fig. 7.

To examine the individual and coordinated effects of facility reconfiguration and signage optimization, three simulation scenarios were evaluated, and the resulting performance metrics are presented in Table 3. Each value reported in the table represents the average result obtained from ten repeated simulation runs for each scenario. The variability across different simulation runs is quantified using 95% confidence intervals, which consistently remain within ± 1% of the mean, indicating good model stability.

Facility reconfiguration alone effectively reduces congestion at major bottleneck facilities, while signage guidance mainly redistributes passenger flows with varying local effects. In comparison, the combined strategy achieves the greatest overall improvement across key performance indicators, demonstrating a clear synergistic effect between capacity enhancement and flow redistribution. In addition, the results presented in the table indicate that the proposed optimization scheme effectively alleviates passenger flow bottlenecks at Entry Gate Group 1 and Ticket Machine Group 1, improving their level of service to Grade C. Despite a reduction in the number of gates, the maximum passenger density at Exit Gate Group 2 increased but remained within an acceptable operational range. This change results from optimized signage guidance that deliberately redirected part of the inbound passenger flow to Entry Gate Group 3, thereby improving overall flow balance and reducing interference between inbound and outbound movements. Although a localized increase in density is observed, this represents a conscious system-level trade-off that contributes to significant congestion relief in key bottleneck areas.

The effectiveness of the proposed optimization scheme primarily stems from two synergistic mechanisms: (1) the reallocation of passenger flow directions via improved guidance signage, which minimizes streamline conflicts and intersection points; and (2) the reconfiguration of in-station facilities, which achieves spatial resource balance and mitigates service pressure in high-density areas. Consequently, the collaborative optimization of guidance and facility layout not only eliminates major bottlenecks but also expands the effective walking area for passengers, thereby enhancing both spatial utilization and passenger flow efficiency within the metro station.

**Table 3:** Comparison of evaluation metrics before and after optimization.

Evaluation metric		Before optimization	Facility-only strategy	Signage-only strategy	After optimization
Maximum Passenger Flow Density (persons/m²)	Entry Gate Group 1	1.09	0.72	0.91	0.62
	Ticket Machine Group 1	1.41	0.69	0.66	0.51
	Exit Gate Group 1	0.50	0.47	0.57	0.47
	Exit Gate Group 2	0.42	0.54	0.32	0.63
Average Queue Length (persons)	Entry Gate Group 1	0.89	0.23	0.28	0.18
	Ticket Machine Group 1	1.79	0.32	0.30	0.13
Average Travel Time (s)	Line 1 Inbound Passenger Flow	295.40	250.18	271.79	218.30
	Line 3 Inbound Passenger Flow	178.28	154.86	166.03	133.52
	Line 1 Outbound Passenger Flow	149.29	150.98	150.26	150.18
	Line 3 Outbound Passenger Flow	165.71	168.71	167.69	168.85

## Discussion

This study addresses the optimization of passenger flow efficiency in subway transfer stations by proposing a passenger flow organization method based on dynamic diversion and synergistic facility configuration. Utilizing an AnyLogic pedestrian simulation system driven by the Social Force Model, the study focuses on peak-hour passenger flows at Subway Station S, quantitatively analyzing the spatiotemporal distribution characteristics of in-station pedestrian density and identifying two critical bottleneck areas: the entry gate group and the ticket machine group. A dual-path diversion mechanism is developed by reconfiguring spatial guidance signage and reallocating ticket machines and gates. Simulation results show that the maximum pedestrian densities at the two bottlenecks decreased by 43.12% and 63.83%, while the average travel times for inbound passengers to Lines 1 and 3 were reduced by 26.1% and 25.1%, respectively. The service level at key nodes improved from Grade F to C. The proposed framework extends the application of the Social Force Model to spatial optimization in transit facilities and provides a quantifiable “signage–facility” collaborative optimization approach for managing high-density passenger flows.

Although the social force model effectively captures individual-level pedestrian dynamics, it exhibits limitations in representing collective decision-making processes, psychological states, and emergency behavioral responses. In addition, to focus on congestion dynamics within the main interchange areas, cross-flows from other functional areas of the station are simplified in the simulation. Consequently, the conclusions of this study are derived from single-station, single-day data and are primarily applicable to regular peak-hour passenger flow scenarios in large interchange stations with relatively stable demand patterns.

Future research will involve collecting multi-day and multi-station data to test the robustness of the optimization results under more complex passenger flow conditions. The problem can also be formulated as a multi-objective optimization framework to systematically search for optimal facility configurations and signage layouts. Furthermore, by incorporating real-time passenger flow data and intelligent algorithms such as machine learning and reinforcement learning, the proposed framework can be extended to support adaptive optimization and dynamic guidance in multi-station and multi-modal transportation hubs, thereby enhancing its robustness, scalability, and decision-support capability for metro station operation and management.

## Data Availability

The datasets generated during the current study consist of simulation data produced by the AnyLogic software based on realistic case scenarios. The AnyLogic simulation model and associated datasets are available from the corresponding author upon reasonable request.
